# Leiomyoma of the prostate: A case report and systematic review

**DOI:** 10.3389/fsurg.2022.878411

**Published:** 2022-07-19

**Authors:** Shangren Wang, Shiqiao Huang, Yang Pan, Yong Ma, Jiaqi Kang, Li Liu, Xiaoqiang Liu

**Affiliations:** ^1^ Department of Urology, Tianjin Medical University General Hospital, Tianjin, China; ^2^ Department of Urology, Shanxian Central Hospital (Affiliated Huxi Hospital of Jining Medical University), Heze, China

**Keywords:** prostate, leiomyoma, surgery, case report, systematic review

## Abstract

**Purpose:**

It is rare to find a large leiomyoma in the prostate, especially in that of a young man. This case report and systematic review provides additional information on the diagnosis, distinguishing features of imaging examinations, and treatment options.

**Patients and Methods:**

We report on the case of a thirty-year-old man with a large leiomyoma of the prostate. MRI of the prostate revealed a round mass in the posterior lobe, 8.0 × 8.0 × 5.5 cm in size. With the assistance of laparoscopy, we resected the prostate mass completely and spared this organ. A systematic review was conducted utilizing the Preferred Reporting Items for Systematic Reviews (PRISMA) including English language published reports, from 1970 to December 2021.

**Results:**

Urinary and erectile functioning was preserved postoperatively. After a year of follow-up, no evidence of recurrence emerged. A total of 21 studies were included for analysis.

**Conclusions:**

A medical history of no, or few, lower urinary tract symptoms; the characteristics of a benign tumor in imaging examinations; and negative tumor markers should be included in any differential diagnosis of leiomyoma of the prostrate. A prostate biopsy should be performed before the preparative radical prostatectomy and choose nonsurgical treatment to confirm the diagnosis. Nowadays, minimally invasive surgery is the preferred effective option for this disease. It is a rare recurrence after its removal by means of surgery.

## Introduction

Leiomymia, most commonly located in the uterus, is a benign tumor composed of smooth muscle fibers (1). A leiomyoma in the prostate is rare, especially a large leiomymia in the prostrate of a young man (2). Leiomyoma of the prostrate may be misdiagnosed as prostate cancer. Treatments for leiomyoma of the prostate have changed in the past decades, and newer treatment options have been applied such as minimally invasive surgery and embolization. Our case and systematic review provides additional information on diagnosis, distinguishing features of imaging examinations, and treatment options.

## Case report

A thirty-year-old man presented at our urology department for a mass in the prostate which had been identified during a routine physical examination. He reported no urinary tract symptoms and no other medical history. On digital rectal examination (DRE), we found a large, solid prostate mass.

Laboratory tests showed a prostate-specific antigen (PSA) level of 1.28 ng/ml (normal 0.00–4.00), a free PSA (f-PSA) level of 0.63 ng/ml (normal 0.00–0.93), a carcinoembryonic antigen (CEA) level of 0.5 ng/ml (normal 0.0–5.0), a carbohydrate antigen 199 (CA199) level of 2.3 U/ml (normal 0.0–37.0), a carbohydrate antigen 50 (CA50) level of 3.5 U/ml (normal 0.0–25.0), an α-fetoprotein (AFP) level of 4.9 ng/ml (normal 0.0–15.0), and a neuron-specific enolase (NSE) level of 6.7 ng/ml (normal 0.0–12.5). The patient had normal renal function, normal liver function and negative urinalysis results.

**Figure 1:**
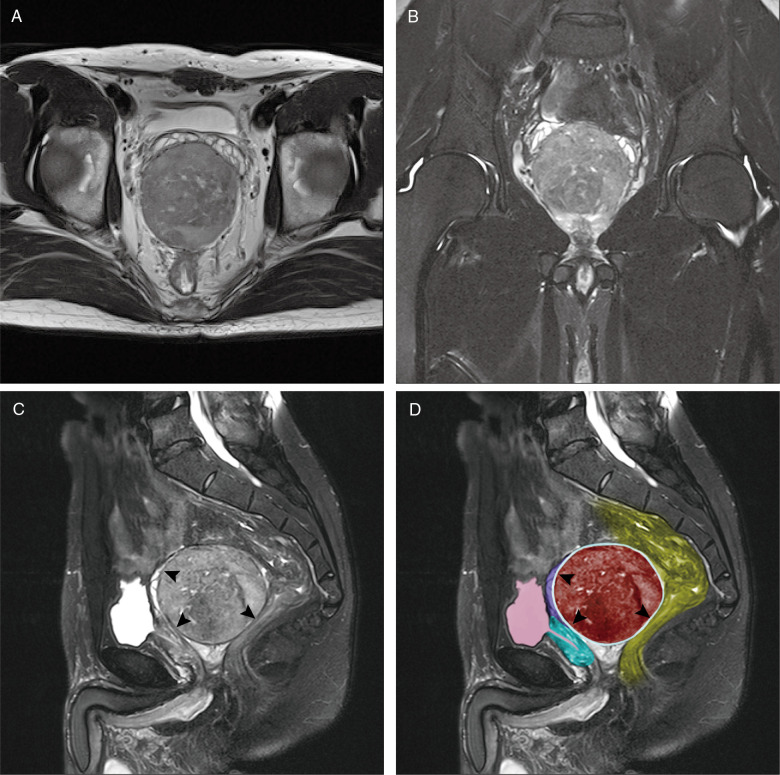
MRI of the leiomyoma of the prostate. MRI T2-weighted axial image (**A**) and fat-suppressed coronal image (**B**) shows a round mass in the prostate posterior lobe; fat-suppressed sagittal image (**C,D**) shows the clear surgical capsule of the tumor (arrow) and shows that normal prostatic glandular tissue and seminal vesicles were compressed anteriorly. The rectum was compressed posteriorly (red: leiomyoma of the prostate; green: prostate; pink: bladder and urethra; yellow: rectum; purple: seminal vesicles; light blue and arrow: surgical capsule of the tumour).

Magnetic resonance imaging (MRI) of the prostate revealed a round mass in the posterior lobe, 8.0 × 8.0 × 5.5 cm in size (Figure 1). With no mass invasion, prostatic glandular tissue and seminal vesicles were compressed anteriorly, and the rectum was compressed posteriorly. A well-circumscribed capsule was observed around the tumor between the normal prostate, seminal vesicles, rectum and other organizational structures (Figure 1). The capsule was clearly visible on MRI axial fat-suppressed T2-weighted images. This capsule was not the tumor's intrinsic capsule; therefore, we referred to it as the surgical capsule (Figure 1C arrow**;** Figure 1D light blue and arrow).

**Figure 2:**
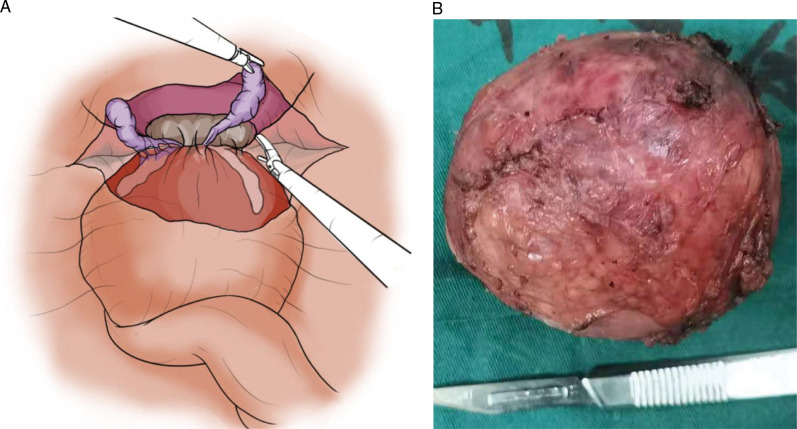
We were able to spare the prostate with the assistance of laparoscopy (**A**) and the prostate mass was completely resected (**B**).

Based on the patient's medical history, PSA and other tumor markers were negative, and MRI revealed a spherically shaped tumor with a surgical capsule. The preoperative diagnosis was a benign prostate tumour. With the assistance of laparoscopy, we resected the prostate mass completely and were able to spare it. We placed three working ports in the abdomen: a 10-mm umbilical port, and 12-mm and 5-mm trocars located at the right and left of the lateral border of the rectus abdominis, respectively. Cavity insufflation of carbon dioxide and abdominal pressure was maintained at 12–14 mmHg. A fifteen degree head-down tilt operating table was used. We found an 8.0 × 8.0 cm solid round mass protruding in the center of the pelvis and outside the peritoneum. The bladder was lifted forward and the peritoneum of the bladder rectal lacuna was opened. The tumor was located between the seminal vesicles and the rectum. The tumor capsule was complete and a clear space (surgical capsular) was not adherent to surrounding organs. The vas deferens, seminal vesicles, and rectum were dissected and protected. Within the surgical capsular space and the external intrinsic capsule of the mass, the tumor was carefully isolated from the prostate tissue with an ultrasound knife, as hemostasis was maintained (Figure 2A). Eventually, the tumor was completely removed (Figure 2B), and the prostate and surrounding structures were spared. A pelvic drainage tube was placed. The amount of intraoperative bleeding was approximately 50 ml and no blood transfusion was required. The surgical procedure was smooth with a duration of 2 h.

**Figure 3:**
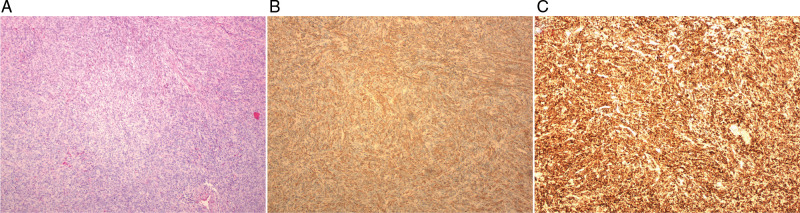
The pathology of leiomyoma of the prostate. (**A**): Histopathological examination showed bland spindle cell proliferation (200×). (**B,C**): Immunohistochemical staining was positive for smooth muscle actin (SMA) (**B**) and Desmin (**C**).

Histopathological examination exhibited a bland spindle cell proliferation (Figure 3A). Immunohistochemical staining was positive for smooth muscle actin (SMA) (Figure 3B) and desmin (Figure 3C), and CD34 (vascular endothelium +, proliferating cells -) was negative for S-100, CD117, Dog-1, CK, and ALK. The Ki-67 proliferation index was low (3%-5%), and the pathological diagnosis was leiomyoma of the prostate.

The patient did not develop any urinary dysfunction after 10 days of surgery and was discharged. During follow-up sessions for a year, the patient's score of International Index of Erectile Function (IIEF 5) (before 23 vs. after 22), International Prostate Symptom Score (IPSS) (before 5 vs. after 2), and Quality of Life (QOL) (before 2 vs. after 2) had no significant difference before and after surgery. Therefore, the patient's urinary function, sexual function and quality of life did not change as compared to before the surgery. And no evidence of recurrence was evident.

## Systematic review

### Methods

A systematic review was conducted utilizing the Preferred Reporting Items for Systematic Reviews (PRISMA) (3). We searched in PubMed, Web of Science databases, Embase, and Medline in the English language from 1970 to December 2021. The keywords used were “Leiomyoma” and “prostate”. All the case reports and series studies were included in this research. All non-English, review articles, for which we could not obtain a full text, were excluded. We extracted the following details of each article: first author, date of publication, country, numbers of patients, age, symptoms, PSA, DRE, the size of the tumor, position, biopsy or not, imaging examinations, initial diagnosis, treatment, follow-up and malignancy.

### Results

**Figure 4:**
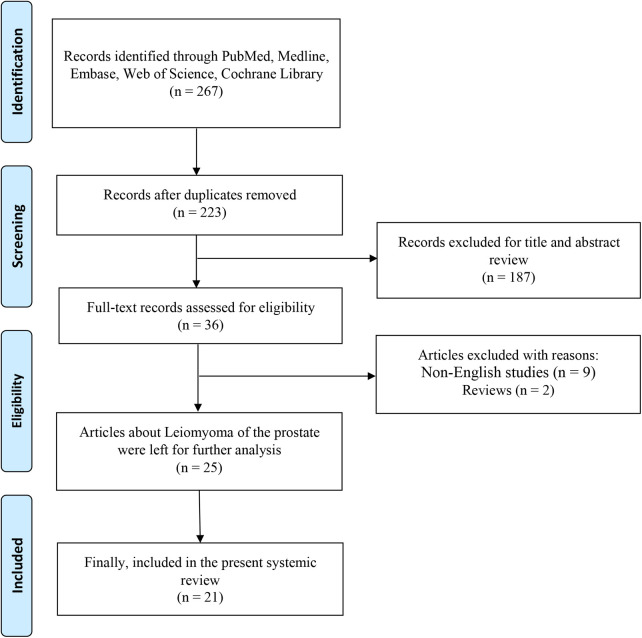
PRISMA flowchart of literature selection process.

After the selection procedure according to PRISMA (Figure 4), a total of 21 studies were included in our systematic review (1, 2, 4, 6–23). There were 35 cases of leiomyoma of the prostate included for analysis and characteristics of the cases are shown in Table 1.

**Table 1:** Characteristics of cases with leiomyoma of the prostate.

Study	Year	Country	Case No.	Age (years)	Symptoms	PSA	DRE	Size	Position	Biopsy	Imaging examination	Initial diagnosis	Treatment	Follow-Up	Malignancy
Albert (6)	1974	USA	1	41	Urinary obstruction	Unclear	Enlarged and firm prostate	Unclear	Unclear	No	IVP: prostatic enlargement	BPH, carcinoma and sarcoma of the prostate	Suprapubie prostatectomy	1 year	No
Cohen (7)	1978	USA	1	65	Urinary obstruction, nocturia.	Unclear	Unremarkable	6 cm	Unclear	No	X-ray and IVP: a mass with calcific encrustations	unclear	Suprapubie prostatectomy	Unclear	No
Vassilakis (8)	1978	USA	1	67	Urinary retention	Unclear	Unremarkable	5 cm	Unclear	No	IVP: a mass	BPH and mass of prostate	Retropubic prostatectomy	Unclear	No
Rosen (9)	1980	USA	1	53	Urinary obstruction, nocturia,	Unclear	Unclear	Unclear	Unclear	No	IVP: normal	BPH	Suprapubic prostatectom	2 years	No
Muzafer (10)	1987	UK	1	78	Painless, intermittent, frank haematuria	Unclear	Unclear	12 cm	Unclear	No	IVP: filling defect in the bladder	Unclear	Transvesical prostatectomy	Unclear	Unclear
Károlyi (11)	1988	Hungary	1	49	Urinary obstruction	Unclear	Enlarged and firm prostate	0.7 cm	Unclear	Yes	Unclear	Unclear	Transvesical prostatectomy	36 months	No
Khalil (12)	1997	UK	1	69	Acute urinary retention	19.3 ng/ml	Unclear	Unclear	Unclear	Yes	Unclear	BPH	TURP	Unclear	Unclear
Yilmaz (13)	1998	Turkey	1	57	Abdominal mass and constipation	Unclear	Prostatic mass	13 cm	Unclear	No	CT: a mass repressed to the bladder and the rectum	Abdominal mass	Suprapubic prostatectomy	Unclear	Unclear
Imai (14)	2002	Japan	1	69	Fever, micturition pain, and testicular swelling	Unclear	Giant prostatic mass	9 cm	Posteroinferior to the bladder	No	CT: well-circumscribed, ovoid mass MRI T1WI: isointense signal relative to the muscle	Prostatic malignant tumor with central necrosis	Suprapubic prostatectomy	Unclear	Unclear
Kitajima (15)	2006	Japan	1	68	Unclear	3.3 ng/ml	Unclear	3.7 cm	Left inner zone	No	MRI: well circumsrcibed nodule	Bladder carcinoma	Radical cystectomy	Unclear	Unclear
Hossain (16)	2008	USA	1	50	Abnormal DRE	Unclear	Abnormal	Unclear	Unclear	Yes	Unclear	Unclear	TURP	6 years	No
			2	59	Abnormal DRE	Unclear	Abnormal	Unclear	Unclear	Yes	Unclear	Unclear	Surveillance	12 years	No
			3	57	Abnormal DRE	Unclear	Abnormal	Unclear	Unclear	Yes	Unclear	Unclear	Surveillance	6 years	No
			4	69	Urinary obstruction	Unclear	Unclear	Unclear	Unclear	No	Unclear	Unclear	TURP	16 years	No
			5	69	Urinary obstruction	Unclear	Unclear	Unclear	Unclear	No	Unclear	Unclear	TURP	4 years	No
			6	80	Urinary obstruction	Unclear	Unclear	Unclear	Unclear	No	Unclear	Unclear	TURP	3 years	No
			7	51	Urinary obstruction and hematuria	Unclear	Unclear	Unclear	Unclear	No	Unclear	Unclear	TURP	9 years	No
			8	82	Urinary obstruction	Unclear	Unclear	Unclear	Unclear	Yes	Unclear	Prostatic cancer	Hormone therapy	4 years	No
			9	66	Urinary obstruction	Unclear	Unclear	Unclear	Unclear	Yes	Unclear	Prostatic cancer	Radical prostatectomy	10 years	No
			10	68	Urinary obstruction, hematuria	Unclear	Unclear	Unclear	Unclear	No	Unclear	Prostatic cancer	Prostatectomy	4 years	No
Mellas (2)	2012	Morocco	1	68	Urinary obstruction	0.3 ng/ml	Enlarged prostate	Unclear	Unclear	No	Unclear	BPH	TURP	Unclear	Unclear
Van (17)	2013	Holland	1	82	Urinary obstruction, hematuria	1.9 ug/L	Enlarged prostate	Unclear	Unclear	No	Urethrocystoscopy and CT: normal	BPH	Retropubic prostatectomy	Unclear	Unclear
Ahmet (18)	2014	Turkey	1	62	Acute urinary, haematuria	3.21 ng/ml	Unclear	Unclear	Unclear	No	Unclear	Unclear	Open prostatectomy	Unclear	Unclear
Ringoir (19)	2016	Belgium	1	54	No symptoms	0.9 mg/L	Enlarged prostate	5.5 cm	Right lobe	Yes	MRI: heterogenous mass with cystic and solid components	Stromal tumor of uncertain malignant potential	Radical prostatectomy	18 days	No
Mussi (20)	2016	Brazil	1	73	Unclear	6.6 ng/mL	Palpable nodule	1.2 cm	Left midgland	Yes	mpMRI: a round nodule with very low signal on T2-WI.	Unclear	Unclear	Unclear	Unclear
			2	60	Unclear	1.7 ng/mL	Abnormal	0.8 cm	Right apex of peripheral zone	Yes	mpMRI: a round nodule with low signal on T2-WI	Unclear	Unclear	Unclear	Unclear
			3	81	Unclear	7.0 ng/ml	Normal	2.8 cm	Periurethral area of the transitional zone	Yes	mpMRI: a poorly-defined lesion, with low signal on T2-WI, restriction diffusion and early enhancement	Unclear	Unclear	Unclear	Unclear
Shen (21)	2017	China	1	76	Urinary obstruction,painful micturition	7.76 ng/ml	Enlarged prostate with no palpable nodule	Unclear	Bottom of prostatic left inner zone	Yes	MRI T2WI: well-circumscribed nodule with a capsule	Prostatic adenocarcinoma	Robot-assisted laparoscopic radical prostatectomy	Unclear	Unclear
Keske (22)	2017	Turkey	1	70	Urinary obstruction	9.8 ng/ml	Unclear	3 cm	Posterior of prostate	Yes	Unclear	Prostatic cancer	Robot-assisted radical prostatectomy	9 months	No
Virarkar (1)	2018	USA	1	56	Urinary obstruction, nocturia	4.1 ng/ml	Enlarged prostate	13 cm	Left lobe	Yes	CT and MRI: a mass arising from the prostate PET-CT: a mass had mild FDG uptake	Leiomyoma	DSA embolization	4 months	No
			2	64	Urinary obstruction	Normal	Unclear	Unclear	Prostate	Yes	CT: large pelvic mass MRI: large, multilobular, heterogeneous pelvic mass PET-CT: not hypermetabolic	Leiomyoma	DSA embolization	4 months	No
			3	51	Nocturia, and urinary frequency	1.1 ng/mL	Prostate nodule	0.8 cm	Left peripheral zone	Yes	TRUS: hypoechoic lesion in the prostate	Leiomyoma	Surveillance	1 year	Unclear
Vergauwen (23)	2018	Belgium	1	62	Unclear	1.05 ng/mL	Unclear	5.8 cm	Right transition zone	Yes	TRUS: an hypoechoic structure MRI: sharply demarcated structure	Benign stromal tumor	Unclear	Unclear	Unclear
			2	74	Urinary obstruction	4.8 ng/mL	Unclear	3 cm	Right inner zone	Yes	MRI: well-circumsrcibed nodule	Unclear	Unclear	Unclear	Unclear
Abeygunasekera (4)	2019	Sri Lanka	1	35	Urinary obstruction	1.1 ng/ml	Enlarged prostate which was firm and having a smooth surface	Unclear	Left lobe	No	TRUS: a mass containing solid and cystic areas. MRI: well-defined solid lesion with multiple cystic areas and surrounded by a hypointense halo	Benign tumour	TURP	Unclear	Unclear

*PSA:prostate-specific antigen; DRE:digital rectal examination; IVP:intravenous pyelography; CT: compute tomography; MRI: magnetic resonance imaging; T1WI: T1-weighted images; T2WI:T2-weighted images; TRUS: transrectal ultrasound; PET-CT: positron emission tomography-CT; BPH:benign prostatic hyperplasia; DSA: Digital subtraction angiography; TURP: transurethral resection of the prostate*.

**Figure 5:**
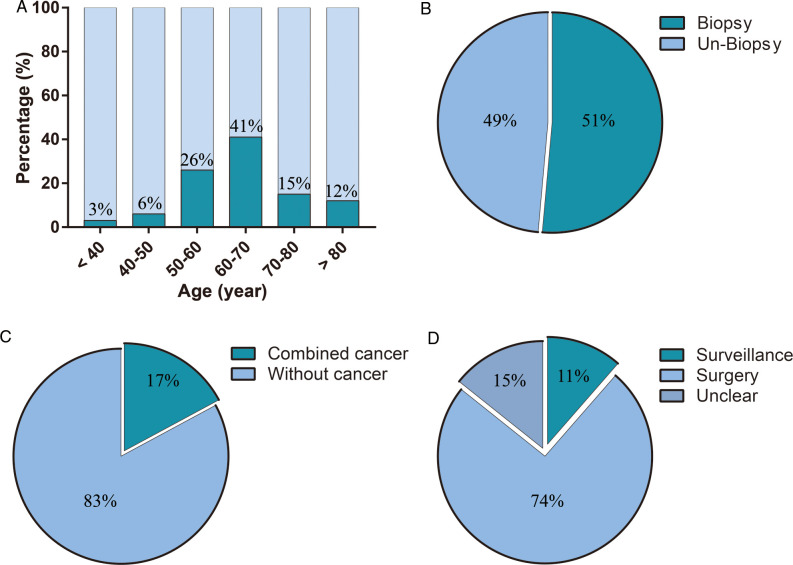
The features of leiomyoma of the prostate, and the experience of diagnosis and treatment. (**A**) age of patients, (**B**) features of biopsy, (**C**) features of combined diseases, (**D**) features of treatment.

**Figure 6:**
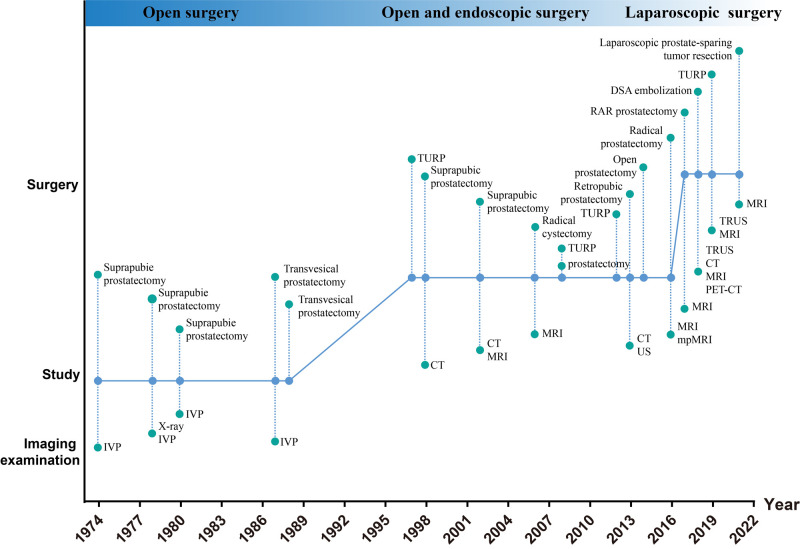
Shifts in the past 50 years of methods of treatment and imaging examination for leiomyoma of the prostate. IVP:intravenous pyelography; CT: compute tomography; MRI: magnetic resonance imaging; PET-CT: positron emission tomography-CT; TRUS: transrectal ultrasound; DSA: Digital subtraction angiography; TURP: transurethral resection of the prostate.

The average age of the patients was 63.86 ± 1.94 years old. The average size (diameter) of the tumors was 5.73 ± 1.03 cm. Leiomyoma of the prostate appeared more frequently in 50–60 year old men (26%) and 60–70 year old men (41%). It rarely occurred in young men <40 years (3%) (Figure 5A). With regard to diagnosis, a biopsy was performed in about 51% cases to obtain a preoperative diagnosis or to distinguish the mass from a malignant prostate tumor (Figure 5B). About 17% cases of leiomyoma of the prostate combined with other malignant tumors, such as prostate cancer or bladder cancer (Figure 5C). For treatment, the majority of cases were subjected to surgical treatment (74%), and only 11% cases were selected for active surveillance **(**Figure 5D). In the past approximately 50 years, the surgical method of choice for leiomyoma of the prostate has gradually changed from open surgery to minimally invasive surgery (Figure 6). Meanwhile, imaging examinations for leiomyoma of the prostate have changed from X-rays or intravenous pyelograms to computed tomography (CT), MRI, and positron emission tomography-CT (PET-CT) (Figure 6).

## Discussion

Leiomyomas are rarely found in the prostate. Kaufman et al. reviewed the relevant literature and first defined the leiomyoma of the prostate as “a circumscribed and encapsulated mass of smooth muscle, 1 cm or more in diameter, containing varying amounts of fibrous tissue but devoid of glandular elements and which is either obviously prostatic or juxta prostatic in origin and position” (5). The exact pathogenesis is still unclear. Inflammatory, infectious and embryological Müllerian duct remnants are the possible mechanisms of pathogenesis (24).

In past decades, some case report studies of leiomyoma of the prostate have been published (1). However, there are still no available guidelines. Imaging diagnoses, preoperative diagnoses, treatment methods and surgical techniques have been insufficiently explored.

We have presented a case of a thirty years old man, who, to our knowledge, is the youngest man suffering from a huge leiomyoma of the prostate in a reported case. In this study, we analyzed the typical imaging features of leiomyoma of the prostate on MRI, and explored prostate-sparing and simple tumor surgical removal techniques. Meanwhile, we performed a systematic review of the English language literature from 1970 to 2021, a period spanning about 50 years, focusing on the features of leiomyoma of the prostate, and the descriptions of diagnosis, imaging examinations and surgical treatments.

### Demographics

Leiomyoma of the prostate occurs more frequently in middle-aged or older men (17). Previous reports have shown that the average age of patients is 63.86 ± 1.94 years old (ranging from 35 to 82 years old). Leiomyoma of the prostate occurs more frequently in 50–60 year old men (26%) and 60–70 year old men (41%). It occurs rarely in young men. In our review, only one case was thirty-five years old (4). In our case report, the patient is a thirty-year-old man. To our knowledge, our case presents the youngest male who has suffered from a leiomyoma of the prostate.

### Diagnosis

Leiomyoma of the prostate has non-specific symptoms. On digital rectal examination, we found a solid prostate mass. Previous reports have shown that lower urinary tract symptoms are common. These include dysuria, urinary frequency, significant nocturia, urgency, painful micturition and acute urinary retention (1, 4, 19). Gross haematuria is a rare symptom, and in our systematic review, only 3 cases reported gross haematuria (10, 17, 18). A large leiomyoma in the prostate posterior lobe would place pressure on the rectum leading to constipation (13).

Leiomyoma of the prostate is sometimes difficult to distinguish from benign prostatic hyperplasia or prostate cancer. In older men, leiomyomas are usually combined with benign prostatic hyperplasia and other malignant tumors, such as prostate cancer or bladder cancer (15, 16). Some leiomyomas of the prostate have been identified in surgical specimens of benign prostatic hyperplasia, prostate cancer and bladder cancer (12, 15).

Few reports have described the features of imaging examinations (14, 15). A hypoechoic or hyperechoic lesion in the prostate may be evident in transrectal ultrasonography (1, 12). A mass of the prostate in the pelvis can be seen in CT imaging (14). These masses are usually well-circumscribed nodules or masses and no lymphadenopathy or distant metastasis may be detected. PET-CT would exhibit no hypermetabolic mass of the prostate (1).

MRI features of leiomyoma of the prostate reveal a well-circumscribed round mass, with isointense and homogeneous signals relative to the muscle on T1WI. A slightly hyperintense or hypointense well-circumscribed mass with a capsule around the tumor between the normal prostate, seminal vesicles, rectum and other organizational structures are exhibited in a T2WI image (23). The capsule is clearly visible in MRI axial fat-suppressed T2-weighted images. It is a feature of a benign tumor. Other traditional and new imaging methods may provide us with more other useful information to make a correct diagnosis, such as TURS, CT, PET-CT.

In our case, the clear surgical capsule around the tumor could be seen in MRI imaging and helped us to make a differential diagnosis from prostate cancer. This capsule was not the tumor's intrinsic capsule. During surgery, along with the capsular space and the external intrinsic capsule of the tumor, we resected the prostate mass completely and were able to spare the prostate. Thus, we referred to it as the surgical capsule.

In general, the PSA of a prostate leiomyoma or leiomyo sarcoma is at a normal level. Abdollahi's study found that PSA has little clinical utility in the diagnosis of leiomyoma (24). When prostate leiomyoma is combined with BPH or prostate cancer, the PSA level becomes raised (12, 20). Biopsy is then useful in assisting with a correct diagnosis. In previous reports, biopsies were performed on about 51% cases before surgery. A preoperative biopsy of the prostate in a small prostate leiomyoma or a prostate leiomyoma of irregular shape is necessary as it is difficult to distinguish between prostate malignant tumors, such as leiomyoma sarcomas, adenocarcinomas and retrovesical ectopic prostatic adenomas (25). We recommend that a prostate biopsy should be performed before the preparative radical prostatectomy to confirm the diagnosis and avoid excessive diagnosis and therapy. And prostate biopsy is necessary for patients who choose nonsurgical treatment to avoid the risk of cancer.

In our case, the PSA was at a normal level, the age of the man was young and on MRI the characteristics of a benign tumor were exhibited. This information did not appear to support a diagnosis of cancer. Nevertheless, we recommend that patients undergo prostate biopsies preoperatively. Moreover, the behavior of the patient was an important factor in our choice of treatment. After communicating with him, he refused to undergo a biopsy as he feared that he would have to wait too long for surgery after the biopsy.

When there is a medical history of no, or few, lower urinary tract symptoms, evident characteristics of a benign tumor in imaging examinations, and negative tumor markers, leiomyoma of the prostate should be included in the differential diagnosis.

The final diagnosis may be reached by histopathological and immunohistochemical analysis (12, 20). The mass of leiomyoma of the prostate is usually well circumscribed, with a smooth surface. It is a homogeneous solid mass with gross features similar to that of a uterine leiomyomas. The mass is composed of bland spindle proliferating cells on microscopic evaluation. No atypical mitosis, pleomorphism or glandular component is identified. In immunohistochemical analysis, the spindle cells are positive for smooth muscle actin (SMA) and desmin. CD34 is negative, in contrast to stromal tumors of unknown malignant potential.

### Treatment

Methods of treatment for leiomyoma of the prostate have changed in the past decades. Prostatectomy was considered the standard surgical approach for leiomyomas of the prostate before the 1990s (10). For aging men, leiomyoma of the prostate frequently combines with benign prostatic hyperplasia. With the development of endoscopic surgery, the transurethral resection of the prostate (TURP) has been gradually applied to the treatment of leiomyoma of the prostate. However, it is important in the case of young men to protect their sexual functioning. At present, with the development of laparoscopy technology, prostate-sparing and simple tumor surgical removal is achievable for pelvic or retroperitoneum tumors (26). However, use of this surgical technique has been rarely reported in studies of leiomyoma of the prostate. In our case, with the assistance of laparoscopy, we resected the prostate mass completely, and the prostate was spared. Furthermore, the urinary and sexual functioning of the patient was preserved after surgery. The key to success in prostate sparing is accurately identifying the surgical capsular space.

Prostate artery embolization is a treatment option for men who are not suited to surgical treatment (1, 23). Active surveillance of asymptomatic patients with a low risk of prostate cancer is necessary. The key to successful nonsurgical treatment lies with the selection of appropriate candidates.

### Prognosis

Leiomyoma of the prostate is a benign tumor. No reports have indicated recurrence of prostatic leiomyomas after surgical removal. In our case, one year after follow-up, there was no evidence of recurrence.

## Conclusion

Leiomyoma of the prostate is a benign tumor. No, or few, lower urinary tract symptoms in the medical history, negative PSA and other tumor markers, and a spherically shaped tumor with a surgical capsule on MRI are indicative of leiomyoma of the prostate, and should be included in relevant differential diagnoses. Other imaging methods may help us with diagnosis, such as TURS, CT and PET-CT. A prostate biopsy should be performed before the preparative radical prostatectomy and choose nonsurgical treatment to confirm the diagnosis. At present, minimally invasive surgery is the preferred effective option for leiomyoma of the prostate as it is important that young men preserve their urinary and sexual functioning. Furthermore, it is a rare recurrence after being completely removed by means of surgery.

## Data Availability

Data available on request from the authors.
